# Reverse Regulatory Pathway (H_2_S / PGE_2_ / MMP) in Human Aortic Aneurysm and Saphenous Vein Varicosity

**DOI:** 10.1371/journal.pone.0158421

**Published:** 2016-06-30

**Authors:** Ingrid Gomez, Gulsev Ozen, Catherine Deschildre, Yasmine Amgoud, Lilia Boubaya, Isabelle Gorenne, Chabha Benyahia, Thomas Roger, Guy Lesèche, Erwan Galardon, Gokce Topal, Marie-Paule Jacob, Dan Longrois, Xavier Norel

**Affiliations:** 1 INSERM, U1148, Paris, 75018, France; 2 University Paris Nord, UMR-S1148, Paris, 75018, France; 3 Istanbul University, Faculty of Pharmacy, Department of Pharmacology, Istanbul, Turkey; 4 UMR 8601, LCBPT, CNRS-Université Paris Descartes, Sorbonne Paris Cité, 75006, Paris, France; 5 AP-HP CHU X. Bichat, Department of Vascular and Thoracic Surgery, University Paris Diderot, Sorbonne Paris-Cité, UMR-S1148, Paris, 75018, France; 6 AP-HP CHU X. Bichat, Department of Anesthesia and Intensive Care, University Paris Diderot, Sorbonne Paris-Cité, UMR-S1148, Paris, 75018, France; Stellenbosch University Faculty of Medicine and Health Sciences, SOUTH AFRICA

## Abstract

Hydrogen sulfide (H_2_S) is a mediator with demonstrated protective effects for the cardiovascular system. On the other hand, prostaglandin (PG)E_2_ is involved in vascular wall remodeling by regulating matrix metalloproteinase (MMP) activities. We tested the hypothesis that endogenous H_2_S may modulate PGE_2_, MMP-1 activity and endogenous tissue inhibitors of MMPs (TIMP-1/-2). This regulatory pathway could be involved in thinning of abdominal aortic aneurysm (AAA) and thickening of saphenous vein (SV) varicosities. The expression of the enzyme responsible for H_2_S synthesis, cystathionine-γ-lyase (CSE) and its activity, were significantly higher in varicose vein as compared to SV. On the contrary, the endogenous H_2_S level and CSE expression were lower in AAA as compared to healthy aorta (HA). Endogenous H_2_S was responsible for inhibition of PGE_2_ synthesis mostly in varicose veins and HA. A similar effect was observed with exogenous H_2_S and consequently decreasing active MMP-1/TIMP ratios in SV and varicose veins. In contrast, in AAA, higher levels of PGE_2_ and active MMP-1/TIMP ratios were found versus HA. These findings suggest that differences in H_2_S content in AAA and varicose veins modulate endogenous PGE_2_ production and consequently the MMP/TIMP ratio. This mechanism may be crucial in vascular wall remodeling observed in different vascular pathologies (aneurysm, varicosities, atherosclerosis and pulmonary hypertension).

## Introduction

Hydrogen sulfide (H_2_S) has recently been identified as gaseous mediator in the cardiovascular system. H_2_S is essentially generated from the endogenous amino acid L-cysteine by the cystathionine-γ-lyase (CSE) enzymatic activity in human and rodent blood vessels [1]. It has been suggested that H_2_S plays a protective role in the pathogenesis and development of cardiovascular disease such as systemic hypertension and atherosclerosis. This gaseous mediator is considered as an oxygen sensor [2] controlling vascular tone and/or vascular wall remodeling. In mice, treatment with sodium hydrosulfide (NaHS, a H_2_S donor), reduced systemic blood pressure [3]. This effect is supported by *in vitro* studies describing relaxation induced by exogenous NaHS in isolated human mammary artery [4] and in vascular preparations derived from different animal species [5]. Furthermore, NaHS decreased the area of atherosclerotic lesions in ApoE knock-out mice [6]. Endogenous H_2_S has been shown to control remodeling of the vascular wall as relative medial thickness of the pulmonary artery could be increased by CSE inhibitors in a model of rat pulmonary hypertension [7].

It has been shown that vascular wall remodeling involved in the pathology of abdominal aortic aneurysms (AAA) and varicose saphenous veins share common determinants, controlled in a reversed manner that results in thinning and thickening of their vascular wall, respectively. The pathogenesis of AAA is characterized by a breakdown of elastic and collagen fibers (the extracellular matrix) due to increased proteolytic activity of serine proteases and matrix metalloproteinases (MMP). The degradation of elastin and of collagens by MMP-1, -2 and -9 plays a major role in vascular wall thinning in AAA [8, 9]. On the contrary, it has been demonstrated that in varicose veins decreased MMP-1 and MMP-2 activities could result in accumulation of collagens and thickening of the vascular wall [10]. There are some studies which have demonstrated an inhibitory role of H_2_S on the expression of MMPs in rat lung [11] and mice brain tissue [12]. However, the significance of H_2_S on MMP expression and activities in varicose vein and AAA pathology, as well as in other human vascular diseases remains undefined.

H_2_S regulates cyclooxygenase (COX) expression and prostaglandin E_2_ (PGE_2_) synthesis. PGE_2_ is a prostanoid produced by nearly all vascular cell types and derives from the arachidonic acid metabolism through the COX and microsomal PGE synthase-1 (mPGES-1). PGE_2_ is degraded by 15-hydroxyprostaglandin dehydrogenase (15-PGDH). The expression of COX-2 and mPGES-1, and in turn the production of PGE_2_, are up-regulated under inflammatory conditions [13]. A number of studies suggest that H_2_S can inhibit COX-2-dependent PGE_2_ production in humans and rodents [14, 15].

It has been shown that EP4 receptors activated by PGE_2_ are involved in the pathogenesis of many vascular diseases, including atherosclerosis [16], AAA [17] and varicose veins [10]. This occurs via the activation of MMP or through regulation of endogenous tissue inhibitors of MMPs (TIMP); EP4 and mPGES-1 expression was found to be lower in varicose veins [10] and greater in AAA [17, 18] compared to healthy vessels. Regulation of MMP/TIMP ratio by PGE_2_ could be responsible for the observed thickening of the vascular wall in varicose veins, and thinning of the structure in AAA.

The entire biological pathway linking H_2_S, PGE_2_ and extracellular matrix expression has never been investigated globally in human vessels. We therefore tested the hypothesis that endogenous H_2_S may modulate PGE_2_, MMP/TIMP ratio and ultimately control the remodeling of the vascular wall in different vascular pathologies, in human varicose saphenous veins and AAA.

## Materials and Methods

### Human vascular preparations

#### Human aorta (healthy and abdominal aneurysm)

All experiments with human subjects were performed in accordance with the Helsinki Declaration of 1975, as revised in 1983. Seventeen healthy aortas (HA; 10 males and 7 females, aged 56 ± 6 years) were sampled either from cardiac transplantation surgery or organ donors with the authorization of the French Biomedicine Agency (PFS 09–007). Seventeen AAA samples (14 males and 3 females, aged 67 ± 2 years) were obtained from patients undergoing surgery, enrolled in the RESAA protocol [19] (*REflet Sanguin de l’evolutivité des Anévrismes de l’Aorte abdominale*, *Comité Consultatif de Protection des Personnes dans la Recherche Biomédical*e, CCPRB Paris-Cochin, approval no 2095). All the aorta samples were without thrombus (luminal layer, at the interface with circulating blood), and adventitia was removed. All patients gave informed written consent and the protocol was approved by a French ethics committee (CPB Paris-Cochin, approval no. 2095, France).

#### Human saphenous veins (healthy and varicose)

Twenty one healthy saphenous veins (SV) from patients undergoing coronary artery bypass surgery (11 males and 10 females, aged 67 ± 5 years) and 23 varicose saphenous veins from patients undergoing vein stripping (11 males and 12 females, aged 61 ± 6 years) were obtained at Bichat Hospital (Paris, France). The varicose veins were obtained from patients at the clinical stage C2 of the disease (CEAP classification of chronic venous disease [20]) without inflammation [21]. Pre-operative evaluation of the vein condition showed retrograde flow but no presence of edema or ulcer. Furthermore, these vessels presented histological features of varicose veins as previously described [20]. All tissues were obtained after patients’ written informed consent. Research programs involving the use of human tissues are supported by the National Institute for Health and Medical Research (INSERM) ethics committee and approved by the French ethics law (L.1211-3–L.1211-9).

After the surgical procedure, the vessels were rapidly transported to the laboratory in ice-cold saline. All tissues were processed within two hours of excision. They were cleaned of adipose tissue and blood, no trace of thrombosis was detectable. Healthy SV used in these experiments were of approximately 3 mm in internal diameter. The varicose veins segments were divided into small diameter varicosity group (SDv) and a large diameter varicosity group (LDv) of approximately 4 mm and 8 mm in internal diameter, respectively. Some fresh vascular samples were incubated for 24h for ELISA measurements (PG, TxB_2_, MMP-1 and TIMPs) or frozen for subsequent analyses (western blot analysis and H_2_S measurement).

### Western blot analysis

Expressions of CSE and mPGES-1 proteins were detected by Western blot. After incubations, the tissue samples were ground in liquid nitrogen and homogenized in RIPA buffer as previously described [21]. Following 15 min of centrifugation at 4500 g, the supernatants were collected and stored at -20°C. Proteins were quantified using a bicinchoninic acid assay kit (ThermoScientific, Rockford, IL, USA). Fifty micrograms of tissue proteins and Western ready controls (CSE ready control from Santa Cruz biotechnology, CA, USA; mPGES-1 ready control from Cayman, Ann Arbour, MI, USA) were submitted to SDS-PAGE and transferred on PVDF membranes. After blocking with PBS 0.5% Tween-20/5% dry milk for 1h, the membranes were probes with a primary antibody, rinsed 3 times for 5 min in PBS /0.5% Tween-20 and incubated with an anti-mouse/rabbit antibody coupled to HRP for 1h (Jackson Immuno Research, West Grove, PA, USA; 1/20,000 dilution). Western blots were revealed by ECL prime (Amersham Biosciences, Glattbrugg, Switzerland). The protein expressions were normalised to beta-actin. The following antibodies were used: CSE (Santa Cruz, 1/5000), mPGES-1 (Oxford Biomedical Research, Oxford, MI, USA; 1/1000), beta-actin (Sigma-Aldrich, Saint-Quentin Fallavier, France; 1/5000).

### Polarographic measurement of hydrogen sulfide production

Endogenous H_2_S production was measured using a polarographic sensor (ISO-H2S-2, WPI) together with an Apollo 1000 free radical analyser (WPI) [22]. The sensor was regularly calibrated with freshly prepared NaHS stock solution using the same buffer and conditions as the experiment. Entire tissue specimens were preincubated for 1 hour at room temperature in 4 ml RPMI solution. Specimens were subsequently placed in the polarographic sensor for a 30 minutes equilibration period in the presence or absence of the CSE inhibitor β cyano-L-alanine at 1 mmol/L (BCA, Cayman, CA, USA). After the equilibration period, L-cysteine was added at incremental concentrations (10 μmol/L, 100 μmol/L, 1 mmol/L, Sigma) every 6–8 min when a plateau was obtained. For basal level of H_2_S measurement, the polarographic sensor was placed alone for equilibration. After 30 minutes, tissues were placed with the sensor for 30 minutes.

### Measurement of prostanoid (PGE_2_, TxB_2_, 15d-PGJ_2_), MMP and TIMP concentrations

Fresh tissue specimens were incubated in RPMI solution (Gibco Invitrogen, Paisley, UK; 166 mg wet weight tissue / mL solution for aorta samples, 70 mg wet weight tissue / mL solution for saphenous vein samples) with 5% CO_2_ for 24h in the absence or presence of NaHS (10 μmol/L, 100 μmol/L, 1 mmol/L, 10 mmol/L; Cayman), of the CSE inhibitor β-cyano-L-alanine (BCA, 10 μmol/L, 100 μmol/L, 1 mmol/L), of PGE_2_ (10 μmol/L, Cayman) or of EP4 receptor antagonist GW627368X (1 μmol/L, Cayman). Incubations with different pharmacological treatments were performed only in venous preparations due to the limited access to aorta samples. RPMI solution always contained antibiotics (penicillin, 1000 IU/mL; streptomycin, 100 μg/mL) and the antimycotic amphotericin (0.25 μg/mL). Supernatants were harvested and frozen until used. The concentrations of PGE_2_, TxB_2_ (stable metabolite of TxA_2_), 15d-PGJ_2_ (stable metabolite of PGD_2_) (Cayman, EIA kits) and MMP/TIMP (DuoSet, R&D systems, Minneapolis, MN, USA, ELISA kits) were measured using an enzyme immunoassay kit according to the manufacturer’s instructions. Results were expressed in pg or ng of tissue wet weight. The concentrations of active MMP-1 were calculated from the measurement of human pro-MMP-1 and total (free and complexed) human MMP-1 using 2 different kits. TIMP-1 and TIMP-2 concentrations were measured using 2 different kits, which recognize the total human TIMP-1 and -2 (free and complexed with MMPs), respectively. Total MMP-2 and MMP-9 (free and complexed) content were quantified by using 2 different kits.

### Data analysis

For protein expression analysis by Western blotting, the optical density (OD) of each band was quantitated from the films using a Scion Image® imaging system. The protein expression detected was normalised to β-actin. For EIA/ELISA measurements, normalisation was performed by correcting for tissue wet weight.

Statistical analysis was performed using the program SigmaStat® (Systat Software, IL, USA). All data are presented as means ± sem derived from (n) patients. Statistical analysis was performed using a two-way ANOVA followed by Bonferroni post-hoc test or a paired t-test between SDv and LDv. P-value<0.05 indicates a statistically significant difference between the compared values. However, in few protocols, when a small number of samples have been used, the results should be taken cautiously.

## Results

### Content of CSE in the saphenous vein and abdominal aorta (healthy and pathological)

**Fig 1:**
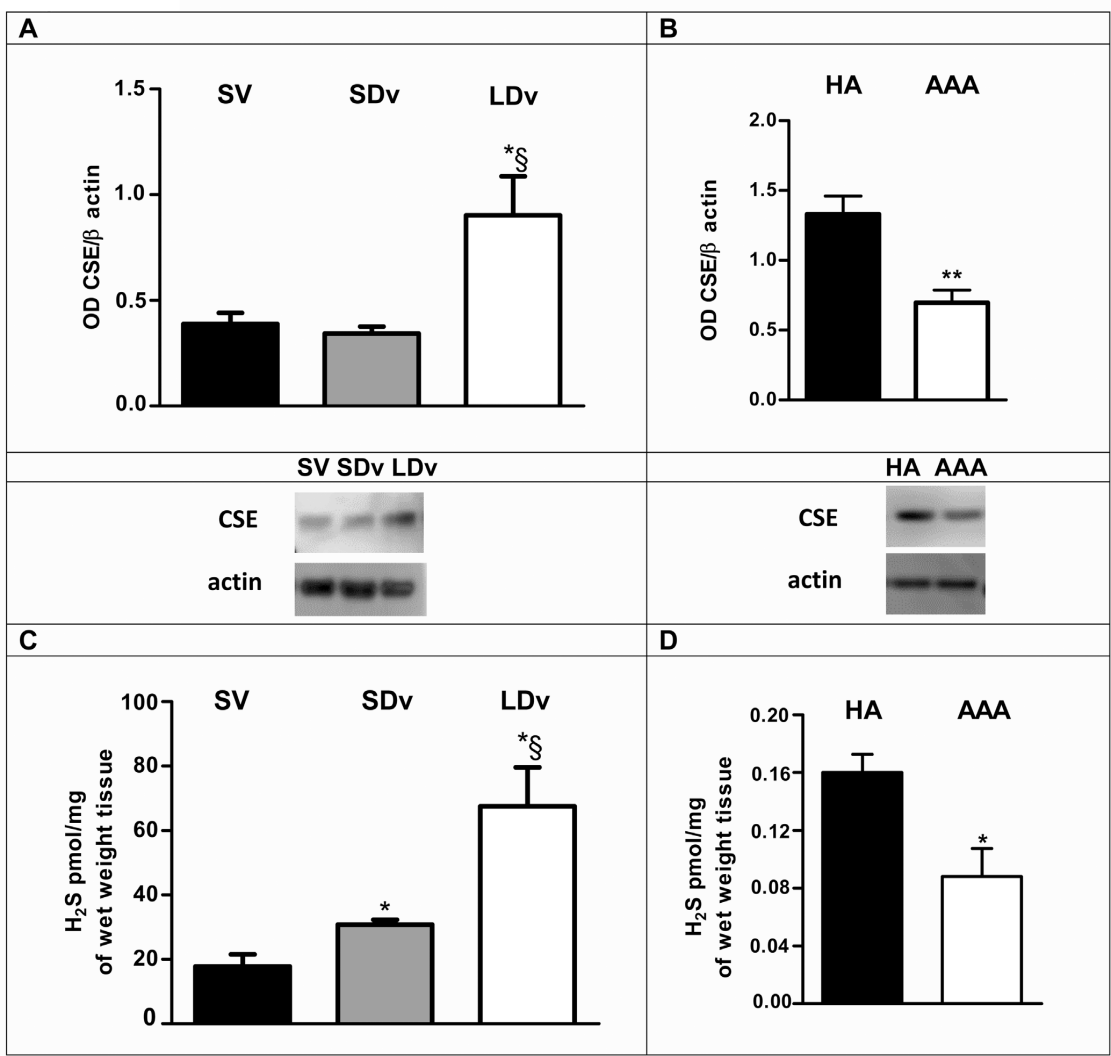
CSE expression and H_2_S production in saphenous veins and abdominal aortas (healthy and pathological). Western blot quantification and representative images of cystathionine-γ-lyase (CSE) normalized to β-actin are shown: **(A)** in healthy saphenous veins (SV, n = 4), in small (SDv) and large diameter (LDv) varicosities (paired n = 4); (**B**) in healthy aortas (HA, n = 5) and abdominal aortic aneurysms (AAA, n = 5). Basal production of hydrogen sulfide (H_2_S) **(C)** by healthy SV (n = 4), varicose veins (SDv and LDv, paired n = 4) and **(D)** by HA (n = 5) and AAA (n = 5) are shown. *P<0.05 versus healthy vessels and §*P*<0.05 between SDv and LDv.

CSE protein corresponding to a 44 kDa band, was found in all vascular homogenates, in SV, in varicose veins (SDv and LDv, Fig 1A), in healthy and pathological aortas (Fig 1B). A significant increase of CSE expression was observed in the LDv samples as compared to SDv and healthy SV. On the contrary, a significant decrease was found in AAA when compared with HA.

### H_2_S production is increased in varicose veins and decreased in AAA

A progressive and significant increase in basal H_2_S production was observed in varicose veins as compared to healthy SV (n = 4; Fig 1C). On the contrary, basal H_2_S production by the media derived from AAA preparations was found significantly lower in comparison to HA (n = 5; Fig 1D).

### Production of H_2_S following L-cysteine addition in healthy and pathological preparations

**Fig 2:**
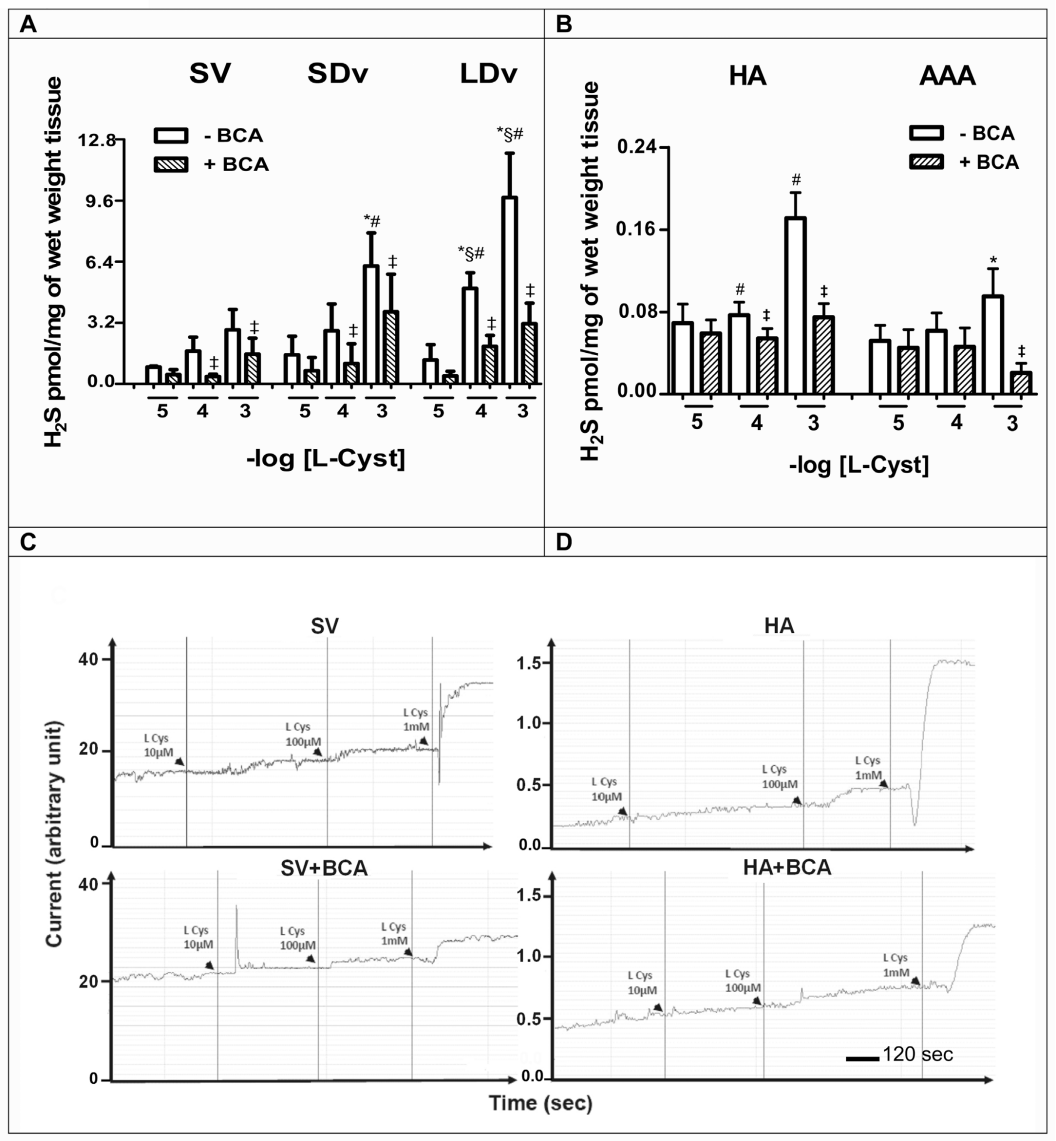
H_2_S production after L-cysteine addition in saphenous veins and abdominal aortas (healthy and pathological). L-cysteine (10^−5^–10^−3^ mol/L) was added in the presence or absence of β-cyano-L-alanine (BCA, 1 mmol/L) in **(A)** healthy saphenous veins (SV, n = 4), small and large diameter varicosities (paired SDv and LDv, n = 4), **(B)** healthy aortas (HA, n = 5) and abdominal aortic aneurysms (AAA, n = 5). *P<0.05 versus healthy vessels or §*P*<0.05 versus SDv treated with the same concentration of L-Cyst; #*P*<0.05 versus L-cysteine 10 μmol/L or ‡*P*<0.05 versus absence of BCA in respective vessels. **(C, D)** Representative traces of production of H_2_S after L-cysteine addition in the presence or absence of BCA in healthy SV and HA using a polarographic sensor.

In all vessels studied, a concentration-dependent production of H_2_S was measured after stimulation with increasing concentrations of L-cysteine (10^−5^–10^−3^ mol/L); these productions were significantly inhibited in the presence of the CSE inhibitor, BCA (1 mmol/L; Fig 2). L-cysteine addition resulted in a significant increase of H_2_S production in LDv as compared to SDv (n = 4) and healthy SV (n = 4; Fig 2A and 2C). On the contrary, H_2_S production was significantly lower in AAA preparations versus HA after addition of L-cysteine (1 mmol/L; n = 5–6; Fig 2B and 2D).

### Variable expression of mPGES-1 in SV and in aorta

**Fig 3:**
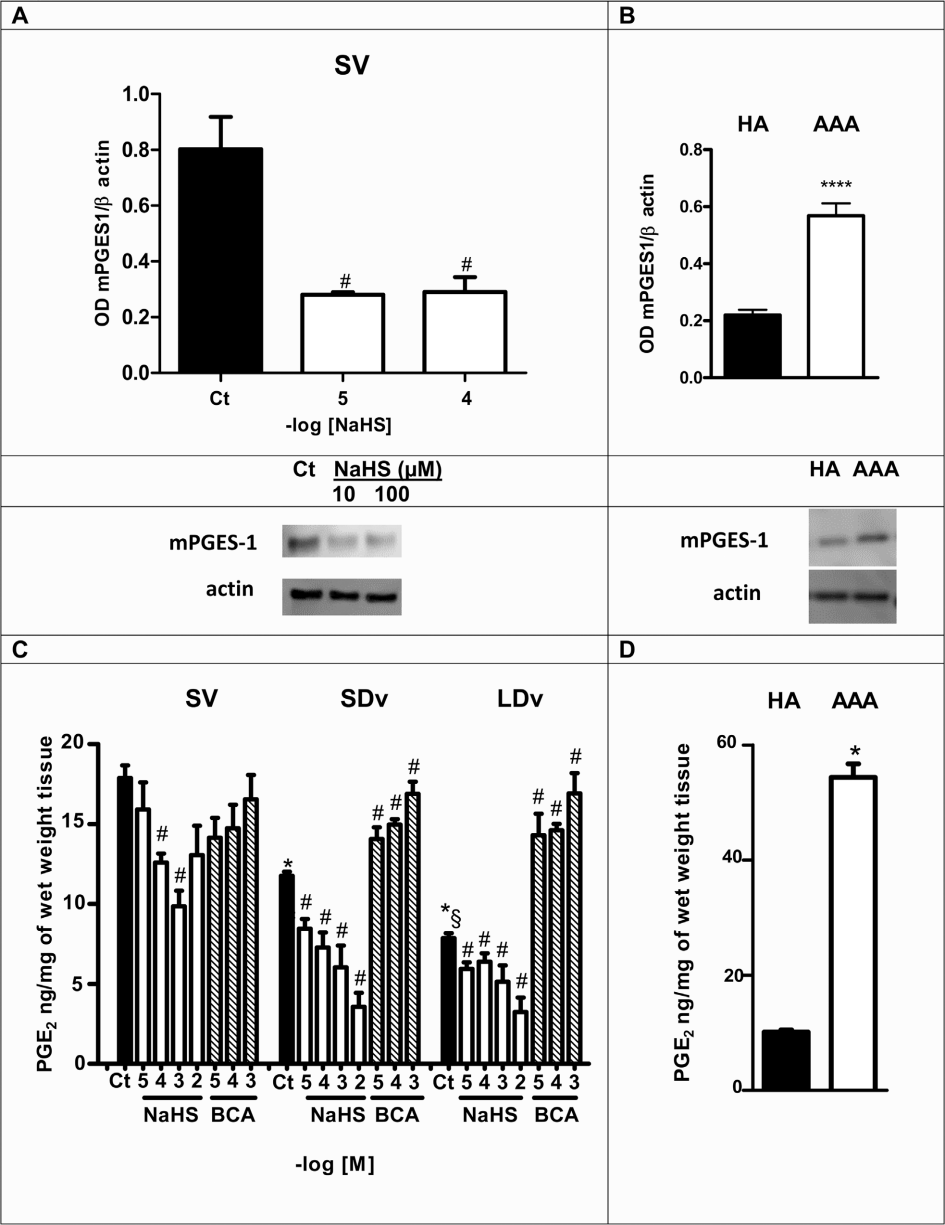
mPGES-1 expression and PGE_2_ production are regulated by H_2_S. Western blot quantifications of microsomal prostaglandin E synthase-1 expression (mPGES-1; normalized to β actin) and representative images are presented. In **(A)** healthy SV untreated (Ct, black bar) or treated (white bars) with NaHS (10^−5^ and 10^−4^ mol/L, 24h, n = 4), in (**B**) healthy aortas (HA, n = 5) and abdominal aneurysmal aortas (AAA, n = 5). PGE_2_ content **(C)** in healthy saphenous veins (SV, n = 14), in small and large diameter varicosities (paired SDv and LDv, n = 14), in **(D)** healthy aorta (HA, n = 3) and abdominal aortic aneurysm (AAA, n = 3) preparations. PGE_2_ concentrations were measured by EIA after 24h incubation with or without the treatments indicated: NaHS (10^−5^–10^−2^ mol/L, n = 4–5) or CSE inhibitor β-cyano-L-alanine (BCA, 10^−5^–10^−3^ mol/L, n = 4–5). *P<0.05, ****P<0.0001 versus respective untreated healthy vessels without treatment; §*P*<0.05 between SDv and LDv; #*P*<0.05 versus respective control.

mPGES-1 protein corresponding to a 16 kDa band, was found in homogenates of healthy SV (n = 4). A significant decrease of mPGES-1 expression was observed in the healthy SV after 24h incubation with NaHS (10^−5^ and 10^−4^ mol/L; Fig 3A). mPGES-1 protein was also found in homogenates of healthy aorta (HA, n = 5) and abdominal aortic aneurysm (AAA, n = 5). A significant increase of mPGES-1 expression was observed in the AAA when compared to HA (Fig 3B).

### PGE_2_ production is decreased in varicose veins and increased in AAA

A progressive and significant decrease in PGE_2_ concentrations in varicose veins (SDv and LDv; n = 14) and a significant increase in AAA preparations (n = 3) were observed in comparison to healthy vessels after 24h incubation (Fig 3C and 3D).

### PGE_2_ production is regulated by H_2_S

After 24h incubation, NaHS (10^−5^–10^−2^ mol/L) significantly decreased PGE_2_ levels in healthy SV (n = 4) and varicose veins (n = 5). In contrast, incubation of varicose veins with BCA (10^−5^–10^−3^ mol/L) reversed significantly the effects of endogenous H_2_S on PGE_2_ (Fig 3C).

### TxB_2_ and 15d-PGJ_2_ productions are not regulated by H_2_S

**Fig 4:**
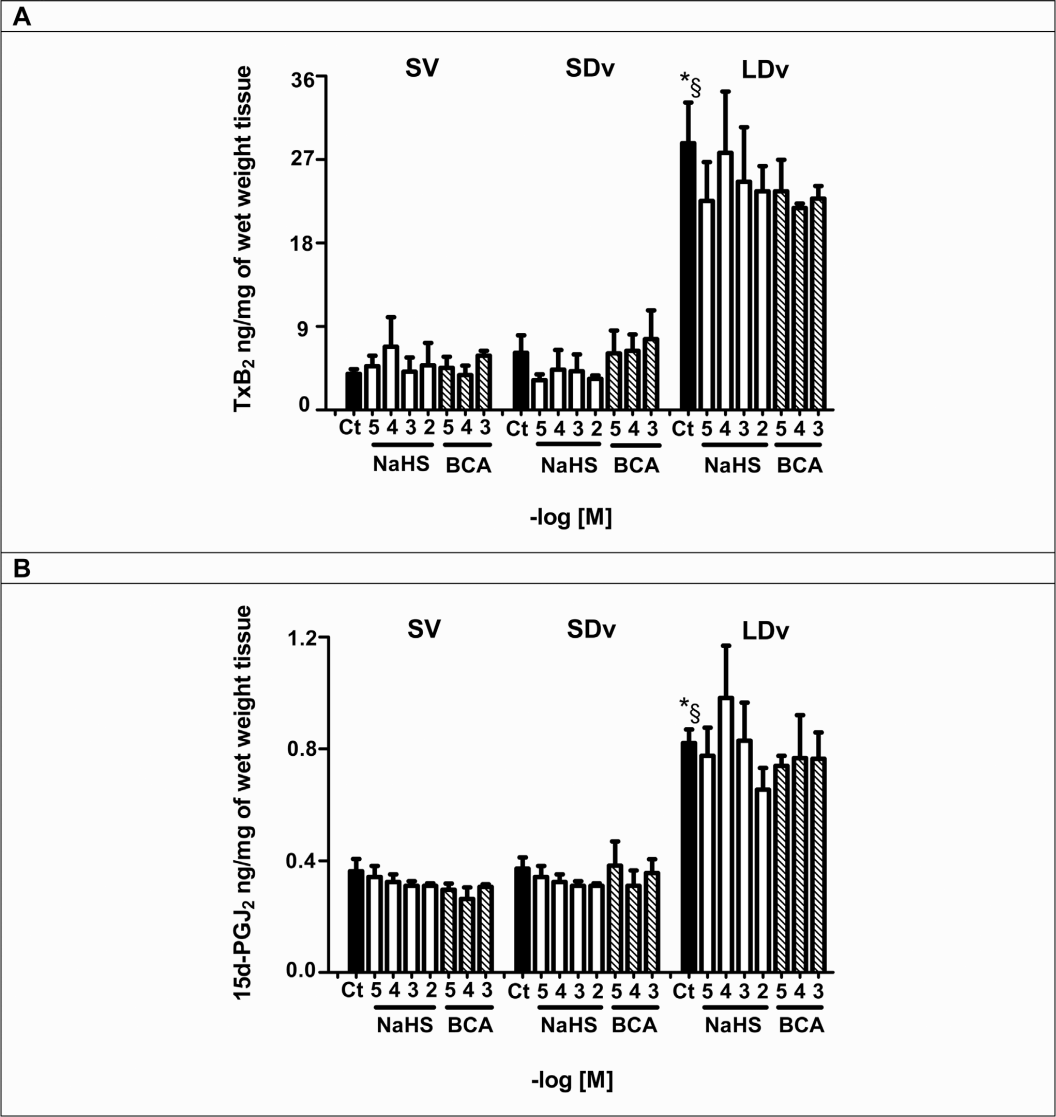
TxB_2_ and 15d-PGJ_2_ productions are not regulated by H_2_S. TxB_2_ **(A)** and 15d-PGJ_2_ **(B)** contents in healthy saphenous veins (SV, n = 4–10) and small and large diameter varicosities (paired SDv and LDv, n = 5–11) were measured by EIA after 24h incubation. Values are normalized by tissue wet weight. Treatments with NaHS (10^−5^–10^−2^ mol/L) and CSE inhibitor β-cyano-L-alanine (BCA, 10^−5^–10^−3^ mol/L) are presented. Ct indicates the respective untreated preparations. *P<0.05 versus healthy SV without treatment. §*P*<0.05 between SDv and LDv.

A significant increase in TxA_2_ and PGD_2_ stable metabolites (TxB_2_ and 15d-PGJ_2_, respectively) productions in LDv (n = 5–11) was found compared to healthy SV and SDv. After incubation with NaHS (10^−5^–10^−2^ mol/L; n = 4) or BCA (10^−5^–10^−3^ mol/L; n = 4) for 24h, no effect on TxB_2_ or 15d-PGJ_2_ release was detected in both healthy SV and varicose veins (Fig 4A and 4B).

### Active MMP-1/TIMPs ratios are decreased in varicose veins and increased in AAA

**Fig 5:**
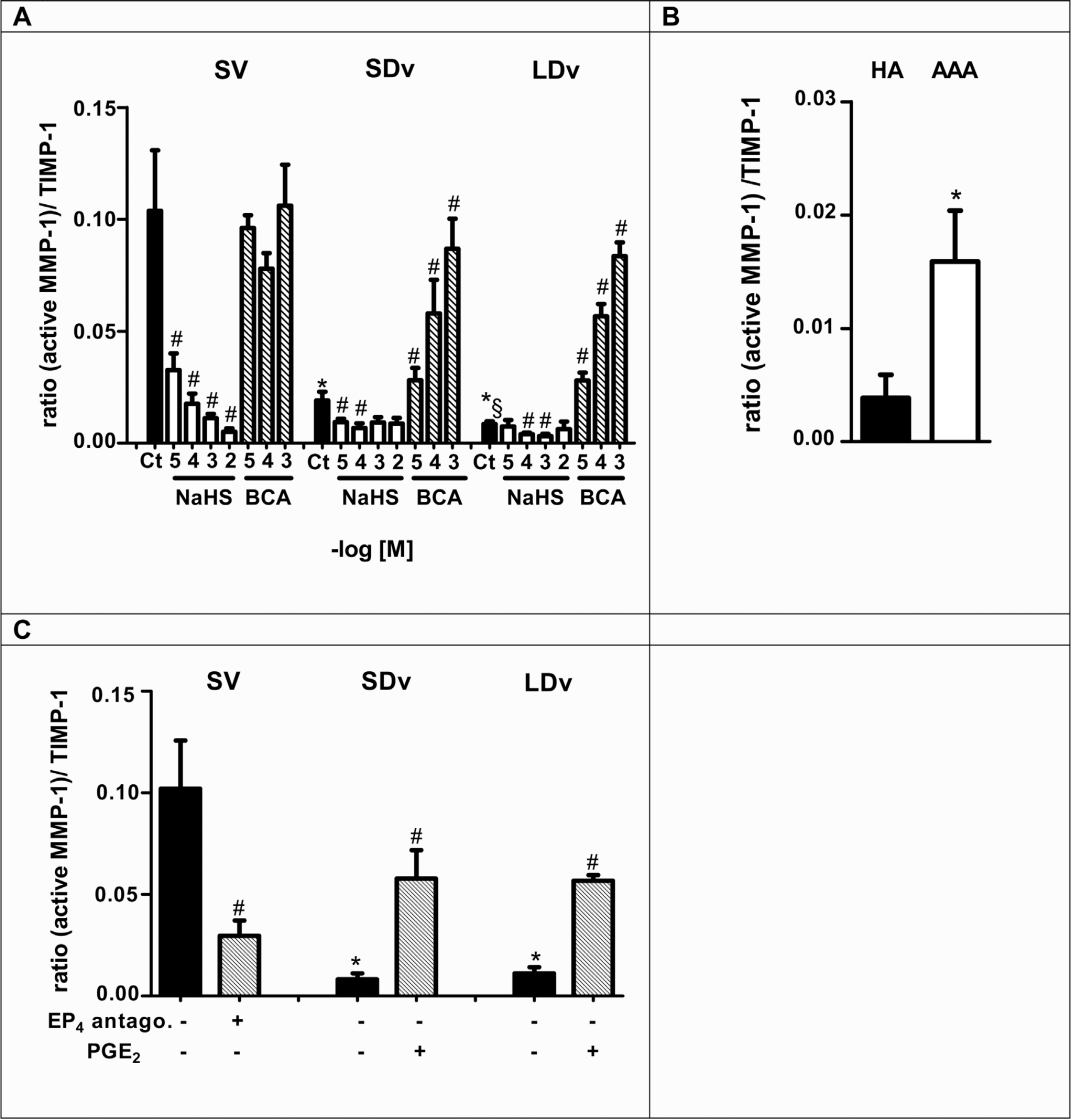
H_2_S is responsible for decreased active MMP-1/TIMP-1 ratio. This ratio was calculated after measurement of active MMP-1 and TIMP-1 contents in healthy saphenous veins (SV, n = 5), small and large diameter varicosities (paired SDv and LDv, n = 6), healthy aortas (HA, n = 5) and abdominal aortic aneurysms (AAA, n = 6–7) by EIA after 24h incubation. MMP-1 and TIMP-1 contents have been normalized by tissue wet weight and ratios are expressed into arbitrary unit. **(A-B)** Treatments with NaHS (10^−5^–10^−2^ mol/L, n = 4–5) or CSE inhibitor β-cyano-L-alanine (BCA, 10^−5^–10^−3^ mol/L, n = 4) are presented. Ct indicates the respective control preparations without treatment. **(C)** PGE_2_ is responsible for active MMP-1/TIMP-1 ratio regulation (n = 4–5). Healthy SV were treated with an EP4 antagonist (GW627368X, 1 μmol/L, 24h), SDv and LDv were treated with PGE_2_ (10 μmol/L, 24h). *P<0.05 versus respective healthy vessel; §P<0.05 between SDv and LDv; #P<0.05 versus respective vessel without treatment.

**Fig 6:**
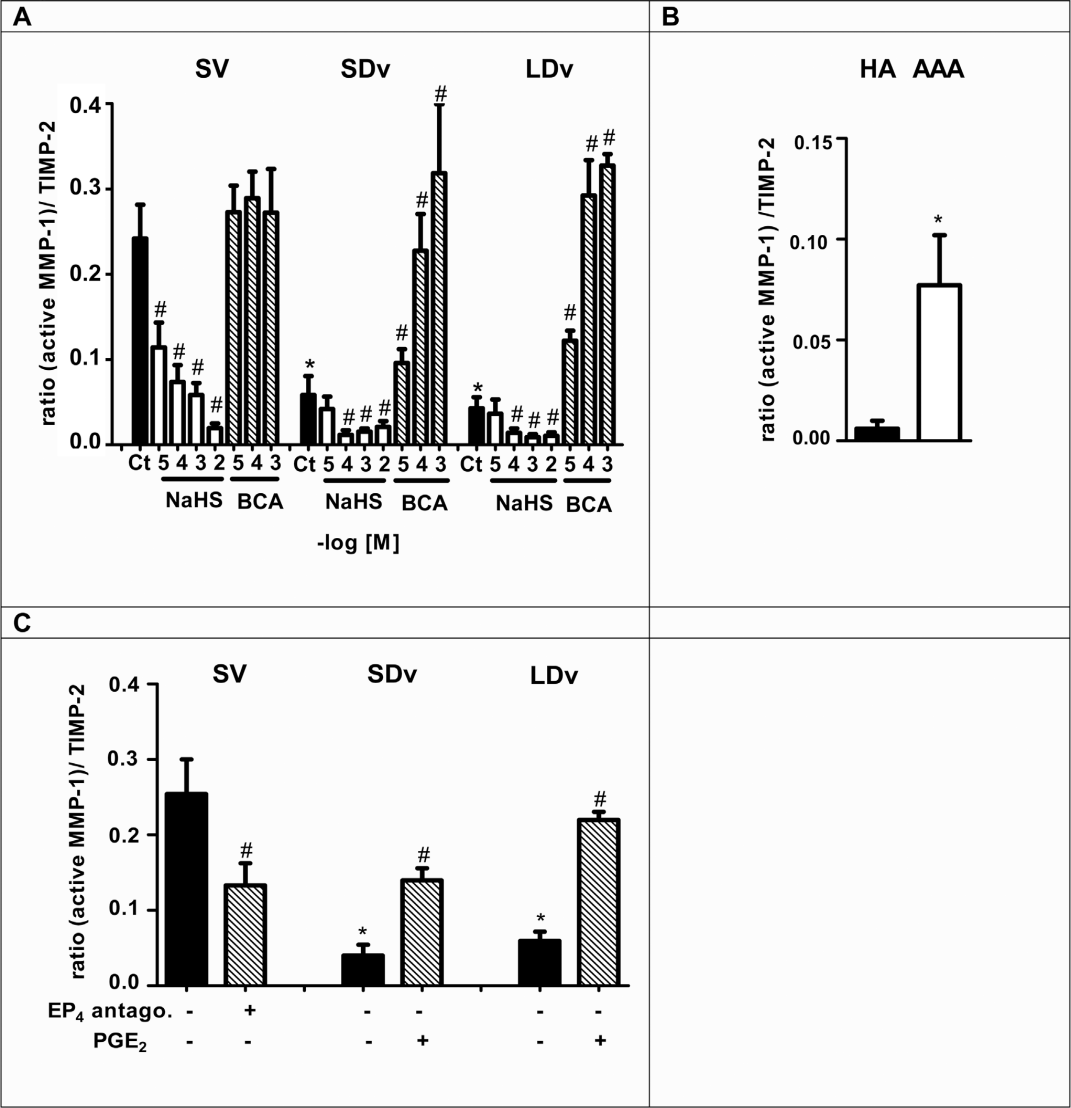
H_2_S is responsible for decreased active MMP-1/TIMP-2 ratio. This ratio was calculated after measurement of active MMP-1 and TIMP-2 contents in healthy saphenous veins (SV, n = 4), small and large diameter varicosities (paired SDv and LDv, n = 5), healthy aortas (HA, n = 5) and aneurysmal aortas (AAA, n = 7) by EIA after 24h incubation. MMP-1 and TIMP-2 contents have been normalized by tissue wet weight and ratios are expressed into arbitrary unit. **(A-B)** Treatments with NaHS (10^−5^–10^−2^ mol/L, n = 3–4) or CSE inhibitor β-cyano-L-alanine (BCA, 10^−5^–10^−3^ mol/L, n = 4) are presented. Ct indicates the respective control preparations without treatment. **(C)** PGE_2_ is responsible for active MMP-1/TIMP-2 ratio regulation (n = 4–5). Healthy SV were treated with an EP4 antagonist (GW627368X, 1 μmol/L, 24h), SDv and LDv were treated with PGE_2_ (10 μmol/L, 24h). *P<0.05 versus respective healthy vessel; #*P*<0.05 versus respective vessel without treatment.

Measurement of active MMP-1, TIMP-1 and TIMP-2 presented Tables 1 and 2 were performed in the same samples used for PGE_2_ measurements after 24h incubation. Increased active MMP-1 levels and decreased TIMP-2 levels were found in AAA preparations compared to HA (Table 2), while exactly the reverse was observed when healthy and varicose veins were compared (Table 1). The ratio of (active MMP-1)/TIMP-1 and (active MMP-1)/TIMP-2 were significantly decreased in varicose veins (n = 6, Fig 5A and 5C and Fig 6A and 6C) and increased in AAA preparations (n = 6–7, Figs 5B and 6B) as compared with their respective healthy vessels (n = 4–5).

**Table 1:** Active MMP-1 and TIMP release from SV, SDv and LDv.

	Active MMP-1	TIMP-1	TIMP-2
Tissue	ng/mg of wet weight tissue	ng/mg of wet weight tissue	ng/mg of wet weight tissue
Healthy SV (n = 15)	0.057 ± 0.006	0.623 ± 0.089	0.231 ± 0.003
SDv (n = 16)	0.017 ± 0.003*	1.130 ± 0.035*	0.351 ± 0.028*
LDv (n = 16)	0.016 ± 0.002*	1.665 ± 0.078*†	0.308 ± 0.017*

MMP: matrix metalloproteinase, TIMP: tissue inhibitor of matrix metalloproteinases, SV: saphenous vein, SDv: small diameter varicosity, LDv: large diameter varicosity.

*P<0.05 versus healthy vessels and

† *P*<0.05 between SDv and LDv

**Table 2:** Active MMP-1 and TIMP release from HA and AAA.

	Active MMP-1	TIMP-1	TIMP-2
Tissue	pg/mg of wet weight tissue	ng/mg of wet weight tissue	ng/mg of wet weight tissue
HA (n = 5)	1.24± 0.67	0.41 ± 0.07	0.16 ± 0.02
AAA (n = 7)	6.62±1.71*	0.44± 0.08	0.10 ± 0.06*

MMP: matrix metalloproteinase, TIMP: tissue inhibitor of matrix metalloproteinases, HA: healthy aorta, AAA: abdominal aortic aneurysm.

*P<0.05 versus healthy vessels

### H_2_S is responsible for decreased (active MMP-1)/TIMP ratio

Exogenous H_2_S led to a significant decrease in active MMP-1/TIMP-1 and MMP-1/TIMP-2 ratios in all segments (n = 4–5). Inhibition of endogenous H_2_S production with BCA induced a significant increase of both ratios in varicose veins versus untreated varicose veins (Figs 5A and 6A).

### PGE_2_ is responsible for increased active MMP-1/TIMP ratio

Exogenous PGE_2_ (10 μmol/L) led to a significant increase of active MMP-1/TIMP-1 ratio (Fig 5C) and active MMP-1/TIMP-2 ratio in varicose veins (n = 5, Fig 6C). The EP4 antagonist (GW627368X, 1 μmol/L) decreased significantly both ratios in healthy SV by inhibiting endogenous PGE_2_ effect (Figs 5C and 6C).

### MMP-2 and -9 are decreased in varicose veins and increased in AAA

**Fig 7:**
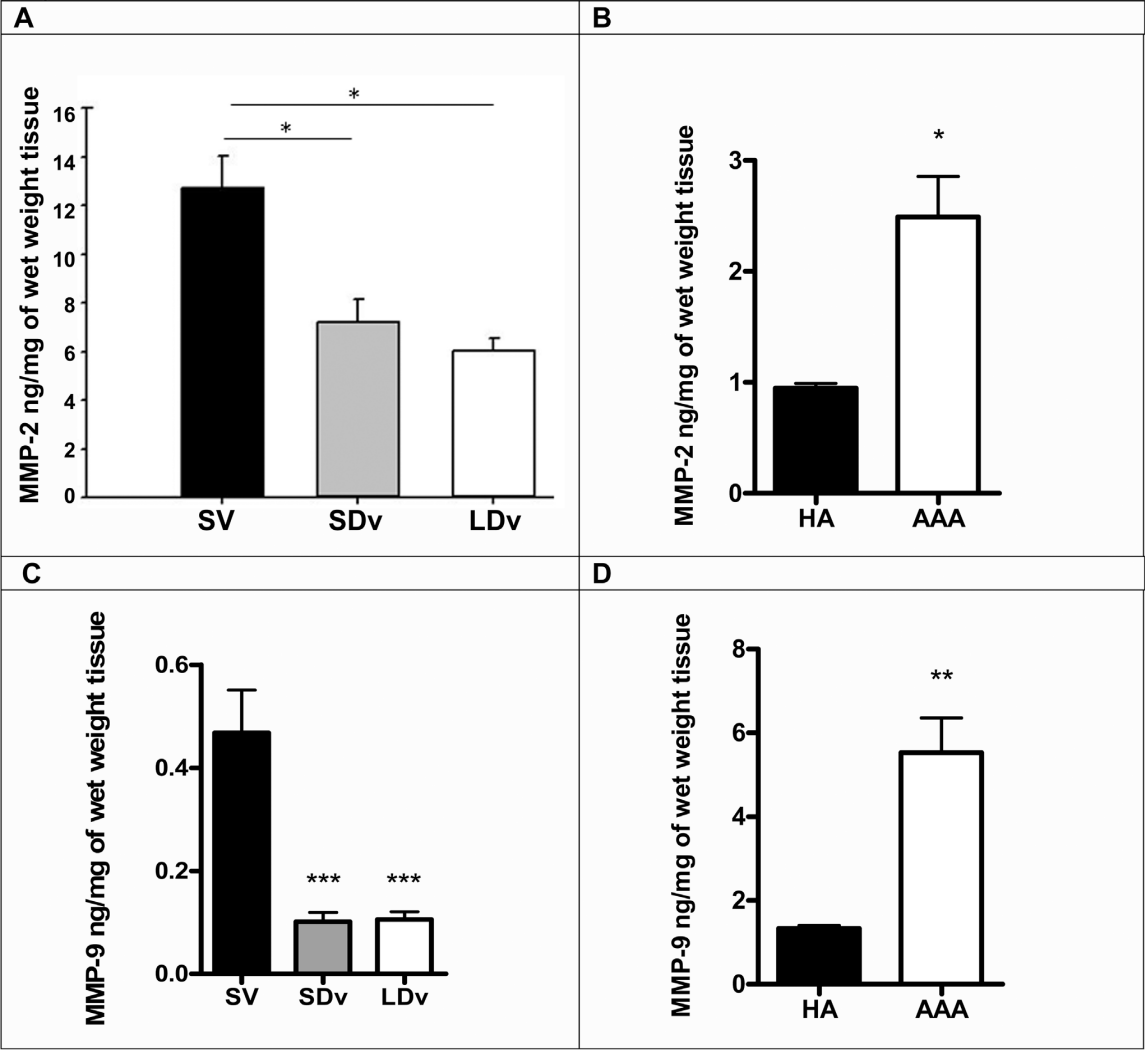
MMP-2 and -9 are decreased in varicose veins and increased in AAA. Measurement of total MMP-2 (**A-B**) and MMP-9 (**C-D**) contents in healthy saphenous veins (SV, n = 5), small and large diameter varicosities (paired SDv and LDv, n = 6), healthy aortas (HA, n = 7) and abdominal aortic aneurysms (AAA, n = 7) by EIA after 24h incubation. MMP-2 and MMP-9 contents have been normalized by tissue wet weight *P<0.05 and **P<0.01 versus respective healthy vessel.

Measurement of total MMP-2 and MMP-9 were performed after 24h incubation. Increased total MMP-2 and -9 levels levels were found in AAA preparations compared to HA (Fig 7B and 7D), while exactly the reverse was observed when healthy and varicose veins were compared (Fig 7A and 7C).

## Discussion

Our results demonstrate that in healthy and pathological human vessels H_2_S and PGE_2_ modulate MMPs involved in vascular wall remodeling. In varicose veins, the increased CSE expression and consequently the increased H_2_S production are responsible for the reduced PGE_2_ content and the reduced MMP/TIMP ratios. In contrast, in medias of AAA, we show a similar cascade of biological events but with a reverse regulation. In consequence, these H_2_S/PGE_2_ regulations are associated with changes in MMP/TIMP ratio and consequently in vascular wall thickening in varicose veins or thinning in AAA.

### Varicose veins

In the present report, H_2_S production was increased in all varicose veins. This is supported by the measurements of CSE content and H_2_S productions in basal conditions or after addition of the CSE substrate (L-Cystein; Figs 1A, 1C and 2A). In addition, the selective CSE inhibitor BCA reduced at least by 50% these responses (Fig 2A) suggesting that H_2_S could also be produced by other enzymes such as cystathionine beta-synthase (CBS) or mercaptopyruvate sulfurtransferase (3-MST) [23].

In the present study, as in our previous report [10], a progressive and significant decrease of PGE_2_ production was observed in varicose veins versus healthy saphenous veins. These new measurements are due to lower mPGES-1 expression in the pathological veins [10]. This decreased expression of mPGES-1 in varicose veins is associated with increased expression of CSE and H_2_S production (Fig 1A and 1C).

Now we show that when venous preparations are incubated with NaHS, mPGES-1 expression and PGE_2_ production are significantly decreased (Fig 3A and 3C). In varicose veins, incubation with BCA restored PGE_2_ content to a level similar to that observed in healthy saphenous veins (Fig 3C). Together these results suggest that endogenous or exogenous H_2_S controls PGE_2_ release in human saphenous vein while other prostanoids release, such as PGD_2_ or TxA_2,_ was not affected (Fig 4A and 4B) suggesting that H_2_S could only effect mPGES-1 expression. The increased levels of PGD_2_ and TxA_2_ (measured by their stable metabolites Fig 4) in varicose veins could be due to increased expression of COX-1 [10]. Previously, it has been shown that a H_2_S donor caused a decrease in COX-2 expression and consequently PGE_2_ levels in human keratinocytes and chondrocytes in inflammatory conditions [24, 25]. More recently, Burguera et al. (2014) have demonstrated that H_2_S donors cause a decrease in PGE_2_ levels by downregulating both COX-2 and mPGES-1 mRNA in interleukin-1beta (IL1β)-stimulated human chondrocytes [14]. Our results are consistent with these previously published results and extend them to the human vascular field.

Even if the pathogenesis of varicose veins is poorly understood, the vascular wall remodeling has been analysed by various studies [26, 27]. Those studies have shown a dysregulation in MMP activities in varicose veins compared to healthy SV. These activities are dependent on MMP activation and concentration of their endogenous inhibitors (TIMP). Increased collagen content has been shown in varicose vein pathology [10], for this reason active collagenase MMP-1 was measured in the present study and we have calculated the active MMP-1/TIMP-1 and MMP-1/TIMP-2 ratios (Figs 5A and 6A). Decreased ratios have been found to correlate with increased collagen content of the venous wall as quantified in our previous report [10]. This result is also supported by the reduced quantities of MMP-2 and -9 measured in varicose veins (Fig 7A and 7C). Furthermore, our pharmacological studies (Figs 5 and 6) show that there is a similar decrease of MMP-1/TIMP ratio after incubation of the healthy saphenous veins with either H_2_S donor or an EP4 antagonist. Conversely, the reduced MMP-1/TIMP ratio measured in varicose veins could be restored (completely or over 50% of the level in healthy saphenous veins), following treatment with a H_2_S synthesis inhibitor or PGE_2_.

Several reports are in accordance with our results obtained in human varicose veins (Table 3). Talaei et al. (2012) showed an inhibitory effect of endogenous H_2_S synthesis on MMP activities (MMP-2, MMP-9) in hamster lung during hibernation [28]. In this study, decreased MMP activity was associated with increased collagen deposition. The H_2_S donors Diallyl(di)sulfide also reduces MMP-2 and MMP-9 activities in human LNCaP prostate cancer cells [29] and in an animal model of lung fibrosis [11]. Similar mechanism could explain the significant decreases of MMP-2 and -9 observed in varicose veins (Fig 7A and 7C). Reversely, in mice cardiac tissues obtained following myocardial infarction, the observed decrease in CSE expression was associated with an increase in MMPs [30]. Other studies have also demonstrated that exogenous H_2_S sources (NaHS) led to reduction of MMP-13 and/or MMP-8 levels in human chondrocytes under inflammatory conditions [14] and in a model of rat heart disease [31].

**Table 3:** Reverse regulatory pathway (H_2_S / PGE_2_ / MMP) in different pathologies.

	CSE	H_2_S	mPGES-1	PGE_2_	15-PGDH	EP_4_	MMP	Collagen Elastin	Consequence on vascular wall
**Varicose Vein**	+ *	+ *	- [10]	- [10]	+ [10]	- [10]	- (MMP-1, -2 [10]; MMP-9 *)	+ Collagen [10]	Thickness [32]
**Pulmonary hypertension**	+ [33]	+ [33]		- [34]	+ [35]			+ Collagen [7]	Thickness [35]
**AAA**	- *	- *	+ [18]	+ [18, 36, 37]		+ [17, 18]	+ (MMP-2 [17]; MMP-1, -9 *	- Elastin [17]	Thinness [38]
**Atherosclerosis**	- [1]	- [6]	+ [16, 39]	+ [39]		+ [39]	+ MMP-1 [40]	- Collagen [40]	Thinness [41, 42] of fibrous cap or media

*Results presented in this manuscript. AAA: abdominal aortic aneurysm, CSE: Cystathionine-γ-lyase, mPGES-1: microsomal PGE synthase-1, 15-PGDH: 15-hydroxyprostaglandin dehydrogenase, MMP: matrix metalloproteinases. (**+**) indicates increase and (-) decrease of expression or density of the molecules.

A similar regulation of collagen accumulation has been observed by Li et al (2007, 2009) who demonstrated that increased CSE and endogenous H_2_S levels in lung tissues of pulmonary hypertensive rat were correlated with increased collagen I and III levels [7, 33]. Thus, the down-regulation of MMP activities by endogenous H_2_S production should increase vascular wall thickness. However, the consequences of MMP regulation are not always negatively correlated to collagen production, some authors showed positive correlations [11, 30] however, these findings are difficult to interpret.

Taken together, these results demonstrate a clear regulatory pathway: high levels of endogenous H_2_S induce a decrease of mPGES-1 expression and PGE_2_ concentration which reduces MMP/TIMP ratio via EP4 receptor activation. This could result in the accumulation of collagen and thickening of vascular wall as it is described either in pulmonary hypertension or in varicose veins (Table 3). Our results support a vasculoprotective role for H_2_S as suggested by a clinical study (thermal hydrotherapy) with sulphurous water in patients with varicose veins [43]. In this treatment, H_2_S has an analgesic effect [44] possibly by reducing PGE_2_ content. However, additionally H_2_S may strengthen the varicose wall by increasing collagen content and so restrain the blood stasis.

### Abdominal aortic aneurysm

To our knowledge, there is currently no evidence regarding H_2_S production and its role in AAA. Studies concerning the effects of H_2_S in human aorta smooth muscle cells (HASMC) suggest that overexpression of CSE stimulates apoptosis due to an increased endogenous production of H_2_S [45]. Furthermore, H_2_S induces hyperpolarization of HASMC and is responsible for relaxation of rat aorta [46].

Our results show for the first time a lower endogenous H_2_S production in AAA compared to HA preparations correlated with a decrease of CSE content (Figs 1B and 1D, 2B and 2D). This reduction was partly due to a reduced CSE activity, since it increased after addition of the substrate of CSE (L-cysteine) and was inhibited by a CSE inhibitor (Fig 2D). This reduction in H_2_S production was associated with increased mPGES-1 content and PGE_2_ synthesis in AAA as compared to HA preparations (Fig 3B and 3D). In accordance with our results, PGE_2_ involvement in the pathogenesis of AAA was observed by other groups [36, 37]. Camacho et al. (2013) showed that mPGES-1/PGE_2_/EP4 axis was up-regulated in human AAA [18]. All these data also are consistent with studies showing that inhibition of mPGES-1 or EP4 expression reduced AAA formation in animal models [47, 48]. Taken together, we suggest a new additional level of regulation of the PGE_2_ pathway by H_2_S in AAA when compared to HA preparations.

We measured active MMP-1 since our study focused on collagen degradation which is required for AAA evolution, even if elastolytic enzymes are important in initiating AAA. The present report shows increased active MMP-1/TIMP-1 and active MMP-1/TIMP-2 ratios in AAA compared to HA. Similarly total MMP-2 and MMP-9 were also increased in AAA. It has been demonstrated that EP4 activation by PGE_2_ is responsible for activation of MMP-2 in human AAA [17]. This activation is in correlation with our data and the increase of total MMP-2 content in AAA (Fig 7B). Yokoyama and coworkers show that this regulation is responsible for decreased elastin levels observed in AAA. This result is in accordance with increased levels of elastin observed in aorta of EP4 knock-out mice and with the negative correlation between EP4 expression versus elastic fiber formation in human AAA preparations [17].

For these reasons, the thinning of human AAA wall may be linked to a reduction in H_2_S production that up-regulates the mPGES-1/PGE_2_/EP4/MMP axis, a reversal of the regulatory processes that result in wall thickening in varicose veins and pulmonary hypertension as previously described.

Our results suggest a clear, common regulatory pathway for the vascular wall remodeling in human AAA and varicose vein pathogenesis. This control is also described in the literature for other vascular pathologies such as atherosclerosis or pulmonary hypertension (see Table 3). This pathway starts from endogenous H_2_S production and regulates *in fine* the collagen content/degradation via intermediary modulation of PGE_2_ and MMP metabolisms. As supported by the literature, the mechanistic behind the effect induced by H_2_S is more probably associated with molecules such as IL-1b and NF-kB. A microarray hybridation study showed that the NF-kB pathway is reversely regulated in human artery and saphenous vein, after OxLDL stimulation [49]. H_2_S protects human H9c2 cardiac cells against high glucose-induced inflammation or against chemical hypoxia-induced injuries, by inhibiting the NF-κB/IL-1β pathway [25, 50]. This down-regulation has been correlated with a decrease of mPGES-1 expression and/or PGE_2_ synthesis in atherosclerotic plaques derived from diabetic patients treated with simvastatin and also in H9c2 cardiac cells [25, 51]. Furthermore, since H_2_S production is intertwined to oxygen partial pressure [2], H_2_S synthesis will depend on the vascular bed and/or the physiopathological state of the vascular wall. In consequence, high or low level of local H_2_S synthesis will result either in thickening (varicose vein) or thinning (aneurysmal aorta) of the vascular wall, respectively.
